# Head-to-head comparison of ^18^F-FDG and ^68^Ga-FAPI PET/CT in common gynecological malignancies

**DOI:** 10.1186/s40644-025-00843-7

**Published:** 2025-02-28

**Authors:** Tengfei Li, Jintao Zhang, Yuanzhuo Yan, Yue Zhang, Wenjie Pei, Qingchu Hua, Yue Chen

**Affiliations:** 1 Department of Nuclear Medicine, Affiliated Hospital of Southwest Medical University, Luzhou, Sichuan 646000 PR China; 2 Nuclear Medicine and Molecular Imaging Key Laboratory of Sichuan Province, Luzhou, Sichuan 646000 PR China; 3 Laboratory for Targeted Radiopharmaceuticals Creation, Luzhou, Sichuan 646000 PR China; 4 Institute of Nuclear Medicine, Southwest Medical University, Luzhou, Sichuan 646000 PR China; 5 Department of Orthopedic, Affiliated Hospital of Southwest Medical University, Luzhou, Sichuan 646000 PR China

**Keywords:** ^18^F-FDG, ^68^Ga-FAPI, PET/CT, Gynecological malignancies

## Abstract

**Background:**

^68^Ga-FAPI (fibroblast activation protein inhibitor) is a novel and highly promising radiotracer for PET/CT imaging. It has shown significant tumor uptake and high sensitivity in lesion detection across a range of cancer types. We aimed to compare the diagnostic value of ^68^Ga-FAPI and ^18^F-FDG PET/CT in common gynecological malignancies.

**Methods:**

This retrospective study included 35 patients diagnosed with common gynecological tumors, including breast cancer, ovarian cancer, and cervical cancer. Among the 35 patients, 27 underwent PET/CT for the initial assessment of tumors, while 8 were assessed for recurrence detection. The median and range of tumor size and maximum standardized uptake values (SUV_max_) were calculated.

**Results:**

Thirty-five patients (median age, 57 years [interquartile range], 51–65 years) were evaluated. In treatment-naive patients (*n* = 27), ^68^Ga-FAPI PET/CT led to upstaging of the clinical TNM stage in five (19%) patients compared with ^18^F-FDG PET/CT. No significant difference in tracer uptake was observed between ^18^F-FDG and ^68^Ga-FAPI for primary lesions: breast cancer (7.2 vs. 4.9, *P* = 0.086), ovarian cancer (16.3 vs. 15.7, *P* = 0.345), and cervical cancer (18.3 vs. 17.1, *P* = 0.703). For involved lymph nodes, ^68^Ga-FAPI PET/CT demonstrated a higher SUV_max_ for breast cancer (9.9 vs. 6.1, *P* = 0.007) and cervical cancer (6.3 vs. 4.8, *P* = 0.048), while no significant difference was noted for ovarian cancer (7.0 vs. 5.9, *P* = 0.179). Furthermore, ^68^Ga-FAPI PET/CT demonstrated higher specificity and accuracy compared to ^18^F-FDG PET/CT for detecting metastatic lymph nodes (100% vs. 66%, *P* < 0.001; 94% vs. 80%, *P* < 0.001). In contrast, sensitivity did not differ significantly (97% vs. 86%, *P* = 0.125). For most distant metastases, ^68^Ga-FAPI exhibited a higher SUV_max_ than ^18^F-FDG in bone metastases (12.9 vs. 4.9, *P* = 0.036).

**Conclusions:**

^68^Ga-FAPI PET/CT demonstrated higher tracer uptake and was superior to ^18^F-FDG PET/CT in detecting primary and metastatic lesions in patients with common gynecological malignancies.

**Trial registration:**

ChiCTR, ChiCTR2100044131. Registered 10 October 2022, https://www.chictr.org.cn, ChiCTR2100044131.

## Introduction

Recent statistics indicate that breast cancer (11.6%), cervical cancer (3.3%), and ovarian cancer (1.6%) are the most commonly diagnosed gynecological malignancies. They also represent the leading causes of cancer deaths among women, accounting for 6.9%, 3.6%, and 2.1%, respectively [1]. Fluorine-18-fluorodeoxyglucose (^18^F-FDG) PET/CT is a valuable imaging modality for the diagnosis, staging, and management of gynecological malignancies; however, certain limitations should be acknowledged [2–4]. ^18^F-FDG PET/CT exhibits low sensitivity in detecting primary lesions and nodal metastases of female cancers due to physiological factors that can lead to variations in ^18^F-FDG uptake. Furthermore, it may not accurately differentiate between acute inflammatory infections and tumor growth [5, 6]. Moreover, ^18^F-FDG uptake can be influenced by blood glucose levels, requiring fasting prior to the ^18^F-FDG PET/CT procedure. This fasting requirement may reduce patient comfort.

Fibroblast activation protein is overexpressed in cancer-associated fibroblasts, which represent the predominant component of the stroma in epithelial neoplasms [7]. Cancer-associated fibroblasts express fibroblast activation protein (FAP), which can be specifically targeted and bound by fibroblast activation protein inhibitor (FAPI) [8]. Additionally, FAP is associated with poor prognosis and the promotion of tumor growth [9]. Consequently, several studies utilizing ^68^Ga-FAPI have been conducted in recent years, yielding promising results across various tumor types and their metastases [10, 11]. ^68^Ga-FAPI PET/CT has shown significant tumor uptake and high sensitivity in lesion detection across a range of cancer types, including head and neck, lung, gastric, colon, and esophageal cancers [12–14]. It is comparable to ^18^F-FDG PET/CT in the diagnosis of primary and metastatic lesions in certain cancer types [15–17]. This study compares the efficacy of ^68^Ga-FAPI PET/CT and ^18^F-FDG PET/CT in detecting primary tumors, lymph node metastases, and distant metastases in common gynecological malignancies.

## Materials and methods

### Patients

This study obtained approval from the Ethics Committee of the Affiliated Hospital of Southwest Medical University (approval no. KY2022114; clinical trial registration no. ChiCTR2200044131). Patients were consecutively recruited for enrollment from January 2022 to December 2023. Both ^18^F-FDG PET/CT and ^68^Ga-FAPI PET/CT were conducted for comparative analysis without affecting patient care. The interval between the two examinations was limited to a maximum of 7 days.

The inclusion criteria were as follows: (a) patients with newly diagnosed or previously treated breast, ovarian, or cervical cancer; (b) patients who underwent paired ^18^F-FDG and ^68^Ga-FAPI PET/CT for tumor staging to determine the most appropriate treatment strategy; (c) patients who underwent paired ^18^F-FDG and ^68^Ga-FAPI PET/CT to detect tumor recurrence and metastases (repeat staging); and (d) patients who provided written informed consent to participate. The exclusion criteria included: (a) pregnant patients; (b) patients with nonmalignant diseases; (c) patients whose treatment commenced prior to their ^68^Ga-FAPI PET/CT examination; and (d) individuals unable or unwilling to provide written informed consent, including research participants, parents, or legal representatives. In this study, histopathologic examination of biopsy or resected surgical specimens served as the reference standard for final diagnosis.

### Acquisition of PET/CT images

For the ^18^F-FDG PET/CT, patients were instructed to fast for 4 to 6 h, and their blood glucose levels were measured to ensure they fell within the reference range of 3.9–6.1 mmol/L. No special preparation was required for the ^68^Ga-FAPI PET/CT examination. The intravenous (IV) doses administered were 3.7 MBq/kg (0.1 mCi/kg) for ^18^F-FDG and 1.85 MBq/kg (0.05 mCi/kg) for ^68^Ga-FAPI. PET/CT scans were conducted approximately 45 to 60 min after IV administration. The CT scan parameters included: tube voltage of 120 kV, current of 120 mA, layer thickness of 3.00 mm, layer spacing of 5 mm, and pitch of 0.813. The PET scan was subsequently performed in 3D acquisition mode on the same table as the CT scan. ^68^Ga-FAPI PET/CT scans were obtained no later than one week after ^18^F-FDG PET/CT, with a median interval of 2 days (range: 1–6 days) between the two examinations.

### PET/CT image analysis

The ^68^Ga-FAPI and ^18^F-FDG PET/CT images were assessed by two board-certified nuclear medicine physicians, and any discrepancies in their interpretations were resolved through consensus. Uptake was classified as positive when an area of focal tracer uptake exceeded the background levels. Furthermore, semiquantitative parameters were calculated using the maximum standardized uptake values (SUV_max_). To minimize bias, studies were reviewed in groups based on their type.

Primary tumors, involved lymph nodes, and distant metastases were classified as positive if their activity surpassed that of adjacent background tissues. Each lesion in the liver, spleen, and bone was recorded individually. Metastases in the peritoneum, mesentery, and omentum were consistently defined as peritoneal carcinomatosis. Tumor size, SUV_max_, median, and range of standardized uptake values were documented.

### Reference standard

All breast lesions, ovarian lesions, and cervical lesions were confirmed by pathology. However, because of technical and ethical issues, pathological findings could not be performed on all suspected involved lymph nodes. We used the results of follow-up CT or MRI as the reference standard. The follow-up time was at least 3 months. During follow-up, the lesion may be considered tumor-related based on remission or progression of suspected involved lymph nodes after anticancer therapy, including chemotherapy, radiotherapy, targeted therapy, and/or immunotherapy.

### Statistical analysis

All statistical analyses were performed using SPSS software (version 20.0; IBM, Armonk, NY). The uptakes of ^18^F-FDG and ^68^Ga-FAPI were compared using the Wilcoxon signed-rank test. The results from visually interpreted PET/CT images were compared with histopathologic findings obtained through biopsy or surgery, which served as the reference standard. The McNemar test was employed to assess the difference in detection rates of primary tumors, lymph nodes, bone metastases, and visceral metastases between ^18^F-FDG and ^68^Ga-FAPI scans. Sensitivity, specificity, and accuracy for both ^18^F-FDG and ^68^Ga-FAPI PET/CT examinations were calculated and compared to evaluate diagnostic efficacy using the McNemar test. A two-tailed P value of < 0.05 was considered statistically significant.

## Results

### Patient characteristics

**Fig. 1:**
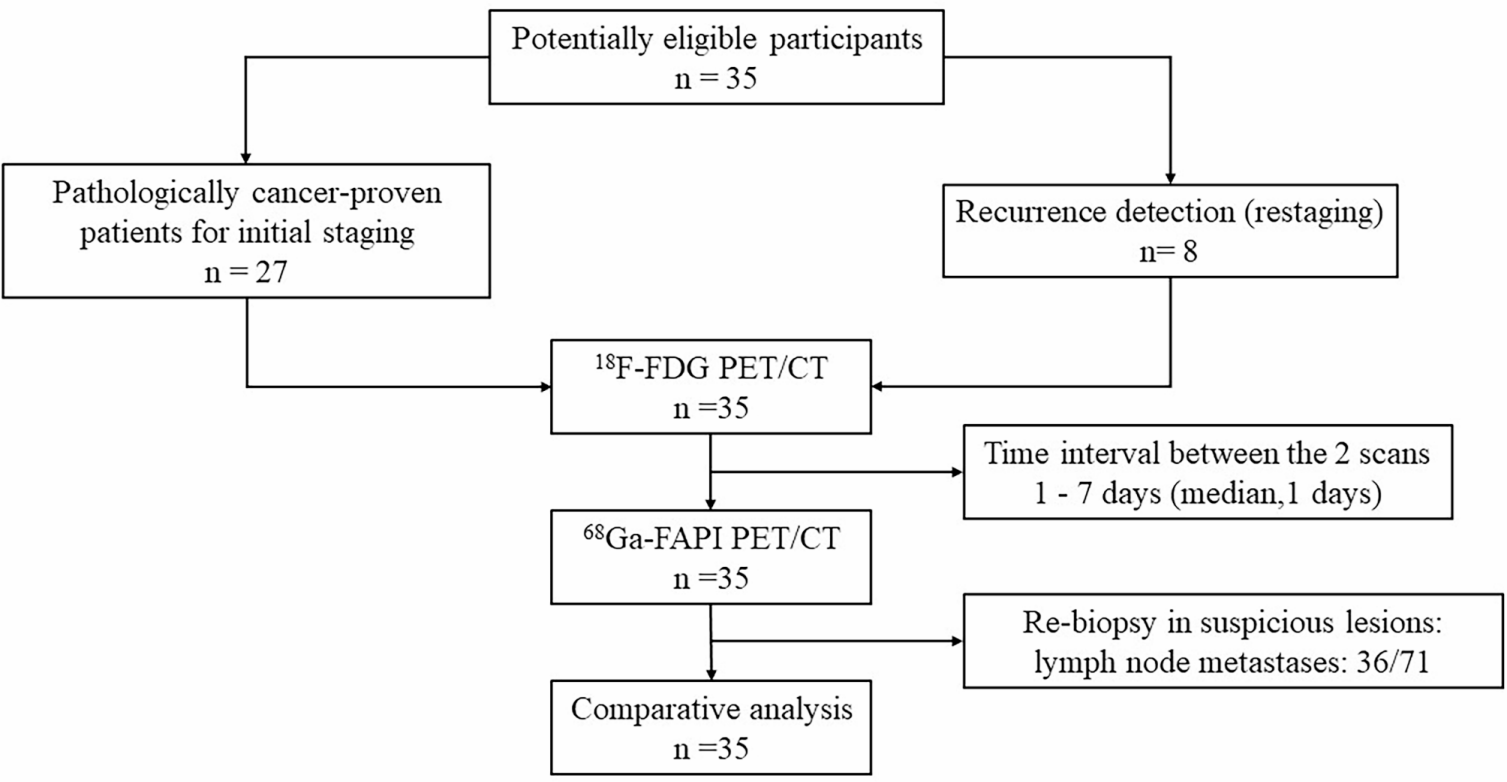
Flow diagram shows patient selection details

This study included 35 female patients (median age, 57 years; interquartile range, 51–65 years) with newly diagnosed or previously treated breast, ovarian, and cervical cancers. The study design is illustrated in Fig. 1. Of the 35 patients, 9 (26%) had breast cancer, 10 (29%) had ovarian cancer, and 16 (45%) had cervical cancer. Within this cohort, 13 patients (37%) were diagnosed with squamous carcinoma, while 22 patients (63%) had adenocarcinoma. A total of 27 patients (77%) underwent PET/CT for the initial assessment of tumors, whereas 8 patients (23%) had PET/CT for recurrence detection. Patient characteristics are summarized in Table 1.

**Table 1:** Summary of patient characteristics

Characteristic	Value
No. of patients	35
Age (y)	
Median	57
Interquartile range	51–65
Diagnosis	
Breast cancer	9
Ovary cancer	10
Cervical cancer	16
Indication for PET	
Initial assessment (staging)	27
Recurrence detection (restaging)	8
Patient status	
Treatment-naive	27
Resection surgery	2
Chemotherapy	3
Chemoradiotherapy	1
Chemotherapy after surgery	1
Chemoradiotherapy after surgery	1
Histologic findings	
Squamous carcinoma	13
Adenocarcinoma	22

### Adverse events

All patients underwent ^18^F-FDG and ^68^Ga-FAPI PET/CT examinations without any adverse events or complications. No signs of pharmacologic effects or physiological responses related to ^18^F-FDG or ^68^Ga-FAPI were observed. Furthermore, none of the patients reported any symptoms.

### Comparative results for initial assessment and recurrence detection

In the assessment of imaging modalities among the 27 patients, ^68^Ga-FAPI PET/CT resulted in upstaging the clinical TNM stage in 5 patients (19%) compared to the stage determined by ^18^F-FDG PET/CT. The confirmed upstaged lesions were verified through CT- or ultrasound-guided biopsy. Among the 8 patients in whom recurrence was detected, the true-positive rates for ^18^F-FDG PET/CT and ^68^Ga-FAPI PET/CT were 50% (4 of 8) and 100% (8 of 8), respectively, on a per-patient basis. The detailed comparative results for initial assessment and recurrence detection are presented in Table 2.

**Table 2:** Comparative results for initial assessment and recurrence detection

A: Initial Assessment									
		Stage with ^18^F-FDG PET/CT				Stage with ^68^Ga-FAPI PET/CT			
Type of Cancer	No. of Patients	I	II	III	IV	I	II	III	IV
Breast cancer	7	1	1	2	3	0	1	3	3
Ovary cancer	7	2	1	2	2	1	1	3	2
Cervical cancer	13	2	2	6	3	1	1	5	6
All	27	5	4	10	8	2	3	11	11
		^18^F-FDG PET/CT				^68^Ga-FAPI PET/CT			
B: Recurrence Detection	No. of Patients	Negative		Positive		Negative		Positive	
Breast cancer	2	1		1		0		2	
Ovary cancer	3	2		1		0		3	
Cervical cancer	3	1		2		0		3	
All	8	4		4		0		8	

### Diagnostic performance of 18F-FDG and 68Ga-FAPI PET/CT in primary tumors

**Fig. 2:**
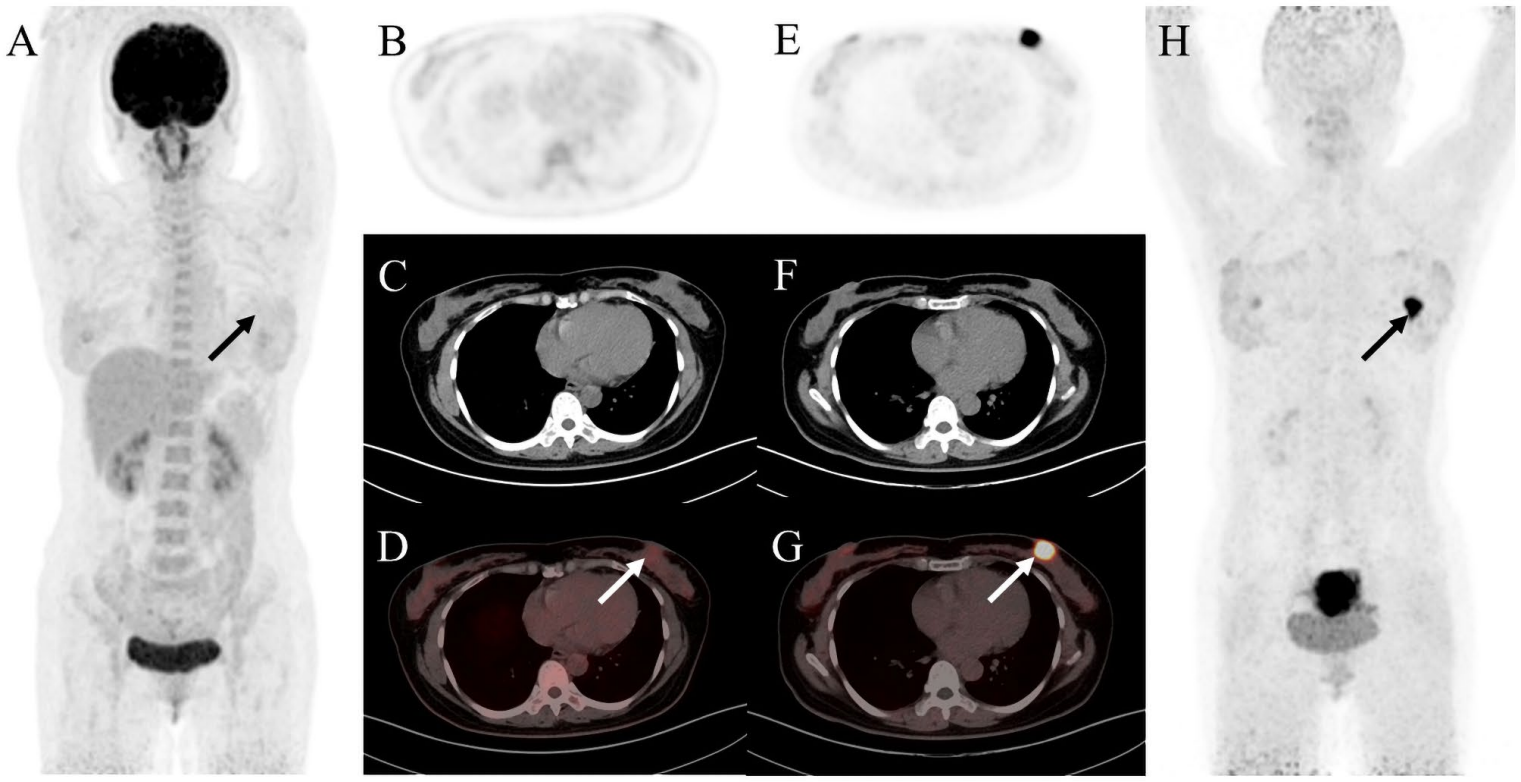
A 47-year-old woman was admitted to our hospital because of left breast mass 20 days ago. For staging, the patient underwent ^18^F-FDG PET/CT. The maximal intensity projection image (**A**) and the axial views (**B**: PET image; **C**: CT scan; **D**: PET/CT fused image) revealed normal findings. Then she was enrolled in our study and underwent ^68^Ga-FAPI PET/CT. The left breast showed intense uptake on ^68^Ga-FAPI-04 PET/CT (**E**: PET image; **F**: CT scan; **G**: PET/CT fused image; **H**: MIP, solid arrow; SUV_max_, 15.4). Subsequently, the patient underwent pathology confirmed invasive lobular carcinoma of the left breast

In evaluating the performance of ^18^F-FDG PET/CT and ^68^Ga-FAPI PET/CT for diagnosing primary tumors in treatment-naive patients, the detection rates were 85% (23 of 27 patients) for ^18^F-FDG PET/CT and 100% (27 of 27 patients) for ^68^Ga-FAPI PET/CT. The false-negative results from ^18^F-FDG PET/CT included breast cancer (*n* = 3) and ovarian cancer (*n* = 1). Notably, cervical cancer had no false-negative detections with ^18^F-FDG PET/CT. Compared to ^18^F-FDG PET/CT, ^68^Ga-FAPI PET/CT exhibited a higher detection rate for primary lesions (100% [27 of 27] vs. 85% [23 of 27], *P* < 0.001) and provided clearer tumor delineation, especially in patients with breast cancer (Fig. 2). Analysis of semiquantitative parameters (Table 3) indicated no significant differences in the primary lesions of breast, ovarian, and cervical cancers between ^18^F-FDG and ^68^Ga-FAPI (7.2 vs. 4.9, *P* = 0.086; 16.3 vs. 15.7, *P* = 0.345; 18.3 vs. 17.1, *P* = 0.703, respectively).

**Table 3:** Comparison of ^68^Ga-FAPI and ^18^F-FDG uptake in common gynecological malignancies

		Tumor Size (cm)		^18^F-FDG Uptake			^68^Ga-FAPI Uptake			
Parameter	No. of Patients	Median	Range	Median SUV_max_	Range of SUV_max_	No. of Positive Lesions	Median SUV_max_	Range of SUV_max_	No. of Positive Lesions	P Value
Primary tumor										
Breast Cancer	7	1.8	0.4–5.3	4.9	2.3–9.8	4	7.2	3.1–15.4	7	0.086
Ovarian Cancer	7	7.1	1.2–13.7	15.7	1.9–22.2	6	16.3	8.5–21.9	7	0.345
Cervical Cancer	13	4.5	2.4–6.3	17.1	7.4–26.3	13	18.3	7.5–26.9	13	0.703
Suspected lymph nodes										
Breast Cancer	9	1.3	1.1–3.7	6.1	5.0-17.2	21	9.9	1.6–22.8	17	0.007
Ovarian Cancer	6	1.2	0.5–1.4	5.9	4.0-13.4	15	7.0	2.2–16.3	12	0.179
Cervical Cancer	6	1.3	0.4–1.5	4.8	3.6–10.4	7	6.3	2.5–16.8	6	0.048
Distant metastases										
Liver	3	2.7	1.3–4.4	7.0	2.4–13.1	13	8.4	4.1–15.2	15	0.109
Peritoneal	4	1.6	0.9–3.2	5.2	2.6–17.4	17	7.1	5.2–12.1	18	0.465
Bone	4	2.4	0.8–3.6	4.9	0.8–6.7	33	12.9	6.5–23.9	43	0.036
Spleen	2	1.1	0.7–2.2	3.1	2.1–4.7	2	5.0	3.7–8.2	3	0.180

### Diagnostic performance of 18F-FDG and 68Ga-FAPI PET/CT in nodal metastasis

The number of positive lymph nodes and the semiquantitative parameters for ^68^Ga-FAPI and ^18^F-FDG PET/CT are presented in Table 3. In the comparison of metastatic lymph nodes detected by both tracers in ovarian cancer, no significant difference in SUV_max_ was observed between the ^68^Ga-FAPI and ^18^F-FDG groups (7.0 vs. 5.9; *P* = 0.179). However, ^68^Ga-FAPI PET/CT exhibited a higher SUV_max_ compared to ^18^F-FDG in breast cancer and cervical cancer (9.9 vs. 6.1; *P* = 0.007; 6.3 vs. 4.8; *P* = 0.048, respectively).

**Fig. 3:**
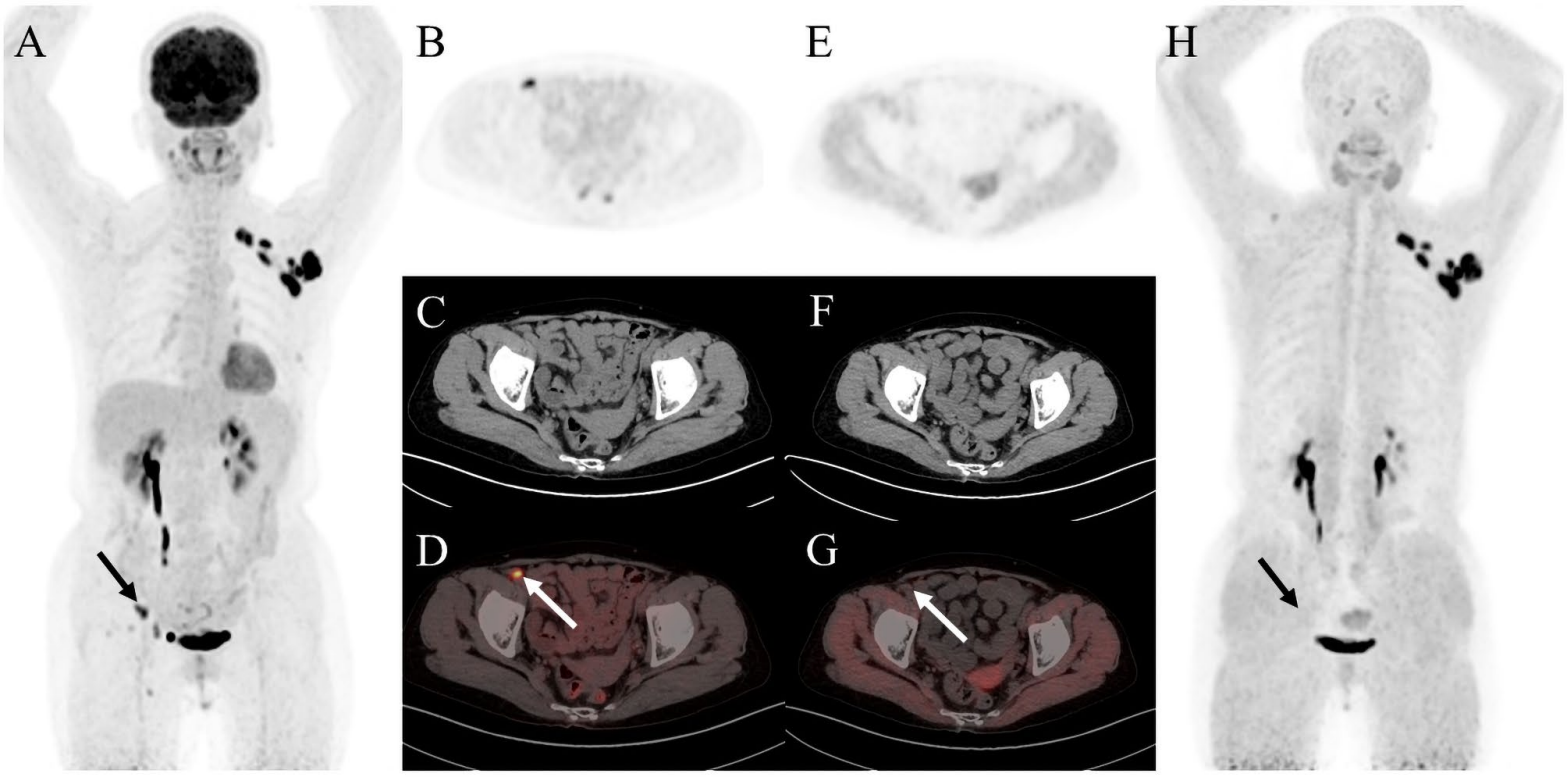
A 68-year-old woman was admitted to our hospital due to the discovery of a left axillary mass for over 10 days. The ^18^F-FDG PET/CT and ^68^Ga-FAPI PET/CT were performed for initial assessment. The MIP image(**A**) and the axial views of ^18^F-FDG PET/CT (**B**: PET image; **C**: CT scan; **D**: PET/CT fused image) showed suspicious lymph nodes in the right external iliac region (solid arrow, SUV_max_, 8.8). However, it showed no corresponding uptake on ^68^Ga-FAPI PET/CT (**E**: PET image; **F**: CT scan; **G**: PET/CT fused image; **H**: MIP). Subsequently, the patient underwent lymph node puncture and pathological results revealed a small number of lymphocytes

A total of 71 suspicious lymph nodes in 21 patients were confirmed through pathological examination (13 via biopsy and 58 via surgical dissection). Among these, metastasis was confirmed in 36 lymph nodes from 12 patients. Lymph node involvement included 35 true-positive, 0 false-positive, 1 false-negative, and 35 true-negative findings using ^68^Ga-FAPI PET/CT, and 31 true-positive, 12 false-positive, 5 false-negative, and 23 true-negative findings with ^18^F-FDG PET/CT. In addition, ^68^Ga-FAPI PET/CT was found to be beneficial in differentiating false-positive lymph nodes (Fig. 3). In the node-based analysis, sensitivity, specificity, and accuracy for diagnosing metastatic lymph nodes were 86% (31 of 36), 66% (23 of 35), and 80% (54 of 71), respectively, for ^18^F-FDG PET/CT, and 97% (35 of 36), 100% (35 of 35), and 94% (67 of 71) for ^68^Ga-FAPI PET/CT (Table 4). The specificity and accuracy of 68Ga-FAPI PET/CT were significantly superior to those of ^18^F-FDG PET/CT (100% [35 of 35] vs. 66% [23 of 35], *P* < 0.001; 94% [67 of 71] vs. 80% [54 of 71], *P* < 0.001, respectively). However, the sensitivity of ^68^Ga-FAPI PET/CT did not exceed that of ^18^F-FDG (97% [35 of 36] vs. 86% [31 of 36], *P* = 0.125).

**Table 4:** Diagnostic performance of ^68^Ga-FAPI and ^18^F-FDG PET/CT in assessment of lymph node metastases

Imaging Modality	Sensitivity (%)	Specificity (%)	Accuracy (%)
^18^F-FDG PET/CT	86(31/36)	66(23/35)	80(54/71)
^68^Ga-FAPI PET/CT	97(35/36)	100(35/35)	94(67/71)
P Value	0.125	<0.001	<0.001

### Diagnostic performance of 18F-FDG and 68Ga-FAPI PET/CT in distant metastases

**Fig. 4:**
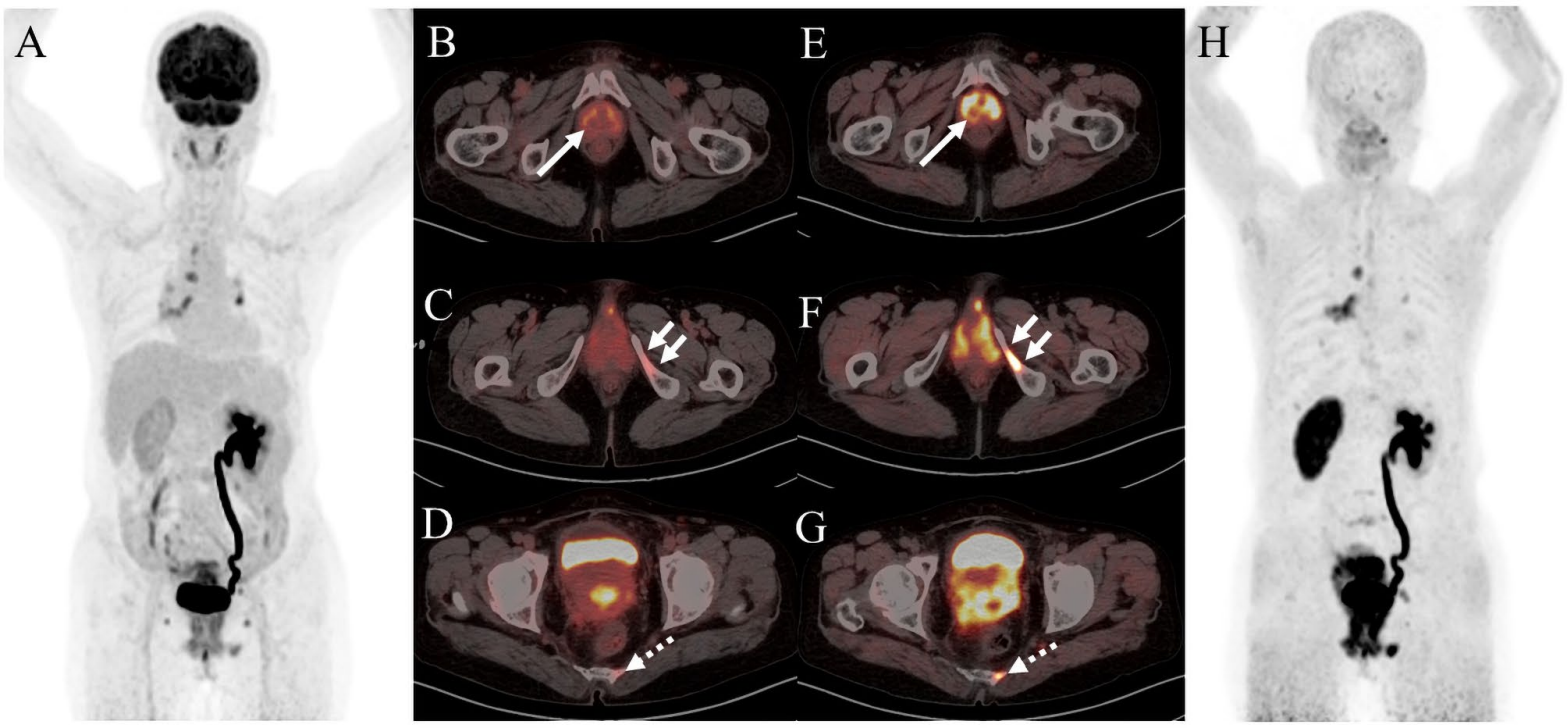
A 63-year-old woman was diagnosed with cervical squamous cell carcinoma in another hospital for more than 3 months and was referred to our hospital. Then ^18^F-FDG PET/CT and ^68^Ga-FAPI PET/CT were performed for initial assessment. The MIP image (**A**) and axial views of ^18^F-FDG PET/CT showed slightly increased uptake in cervix (**B**, solid arrow; SUV_max_, 8.6), left sacrum (**C**, solid arrow; SUV_max_, 2.4), and left ischium (**D**, dashed arrow; SUV_max_, 0.8). While ^68^Ga-FAPI PET/CT revealed significant increased FAPI uptake in corresponding cervix (**E**, solid arrow; SUV_max_, 12.5), left sacrum (**F**, solid arrow; SUV_max_, 11.1), and left ischium (**G**, dashed arrow; SUV_max_, 7.0). Subsequently, the patient received radiotherapy. It showed the left sacrum and left ischium lesion decreased in size after 3 months

**Fig. 5:**
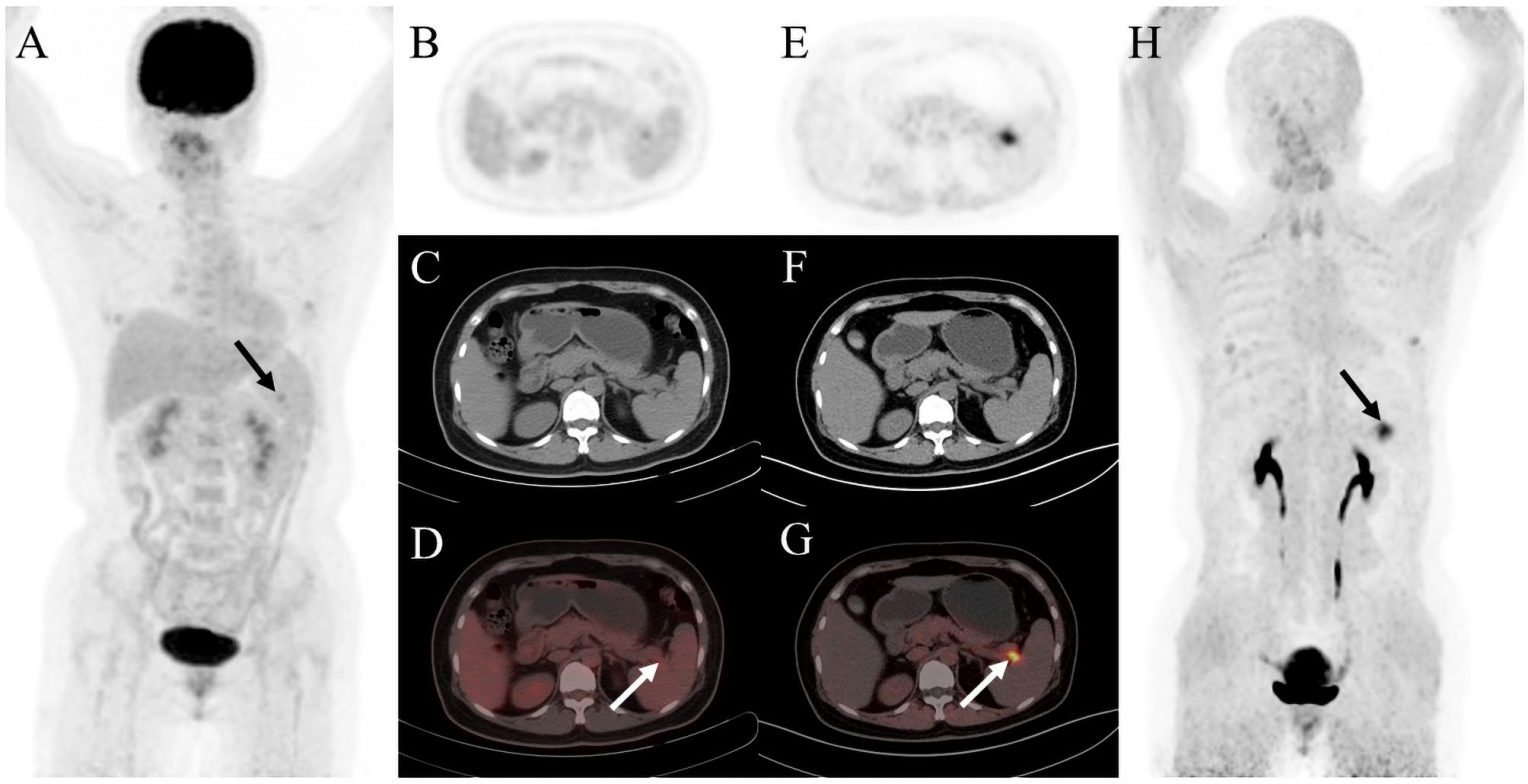
A 45-year-old woman was admitted to our hospital because of bleeding after intercourse for one month. A subsequent cervical biopsy indicated cervical adenocarcinoma. For staging, the patient underwent ^18^F-FDG PET/CT. The MIP image (**A**) and the axial views (**B**: PET image; C: CT scan; **D**: PET/CT fused image) revealed no abnormal uptake. Subsequently, ^68^Ga-FAPI PET/CT was performed. Spleen with intense uptake (**E**: PET image; **F**: CT scan; **G**: PET/CT fused image; H: MIP, solid arrow; SUV_max_, 8.2) was observed on ^68^Ga-FAPI PET/CT. Subsequently, spleen metastasis was confirmed by pathological result

Table 3 presents the number of positive metastatic lesions and the semiquantitative parameters for ^68^Ga-FAPI and ^18^F-FDG PET/CT. The SUV_max_ of ^68^Ga-FAPI did not differ significantly from that of ^18^F-FDG in most distant metastases (liver metastases: 8.4 vs. 7.0, *P* = 0.109; peritoneal metastases: 7.1 vs. 5.2, *P* = 0.465; spleen metastases: 5.0 vs. 3.1, *P* = 0.180). However, for detecting bone metastases, ^68^Ga-FAPI exhibited a higher SUV_max_ than ^18^F-FDG (12.9 vs. 4.9, *P* = 0.036). Moreover, ^68^Ga-FAPI PET/CT depicted more metastatic lesions and higher SUV_max_ compared to ^18^F-FDG PET/CT, particularly in bone (Fig. 4) and spleen (Fig. 5) metastases.

## Discussion

Gallium-68 (^68^Ga)-labeled fibroblast activation protein inhibitors (FAPIs) are novel radiotracers designed to target FAP. These ^68^Ga-FAPIs enable visualization of the stroma in epithelial neoplasms, offering a promising alternative to fluorine-18 (^18^F) fluorodeoxyglucose (FDG). Our study demonstrated that ^68^Ga-FAPI PET/CT is more sensitive than ^18^F-FDG PET/CT for identifying primary tumors in breast, ovarian, and cervical cancers (100% [27 of 27] vs. 85% [23 of 27], *P* < 0.001). Regarding the diagnostic performance for nodal metastasis, ^68^Ga-FAPI PET/CT shows significant advantages in specificity and accuracy (100% [35 of 35] vs. 66% [23 of 35], *P* < 0.001; 94% [67 of 71] vs. 80% [54 of 71], *P* < 0.001). However, no significant difference was found between ^68^Ga-FAPI PET/CT and ^18^F-FDG PET/CT in the sensitivity of detecting lymph node metastases (97% [35 of 36] vs. 86% [31 of 36], *P* = 0.125). In terms of diagnostic performance for distant metastases, ^68^Ga-FAPI PET/CT demonstrates a higher SUV_max_ compared to ^18^F-FDG PET/CT in bone metastases (12.9 vs. 4.9, *P* = 0.036).

Previous studies have shown intermediate expression of fibroblast activation protein (FAP) in ovarian and cervical cancers, along with high expression levels in breast cancer [10, 12]. These findings align with our results; the uptake of ^68^Ga-FAPI in primary tumors of breast, ovarian, and cervical cancers was high. Furthermore, elevated FAP expression in breast and ovarian cancers appears to correlate with advanced tumor grades and poorer prognosis [18, 19]. Our study indicates that five patients were restaged to a different clinical TNM stage based on the results of ^68^Ga-FAPI PET/CT. In addition, FAPI uptake appears to increase in hormone-responsive organs, such as the breast, during lactation and following hormonal stimulation, as observed in two individual case reports [20, 21]. Furthermore, a retrospective analysis of 77 female patients indicated that physiological uptake may limit the diagnostic value of FAPI in uterine body malignancies [22]. This variability in uptake, influenced by hormonal factors, may pose challenges to the diagnostics of gynecological malignancies using FAPI. Remarkably, the single-molecule FAPI serves as both a diagnostic and potentially therapeutic agent, facilitating additional theranostic applications [8, 23].

Precise staging and lymph node detection are essential for the treatment and prognosis of cancer patients [24–27]. In our study, the uptake of ^68^Ga-FAPI in metastatic lymph nodes associated with breast and cervical cancers exceeded that of ^18^F-FDG. Furthermore, ^68^Ga-FAPI PET/CT may offer greater specificity and accuracy than ^18^F-FDG in detecting metastatic lymph nodes in patients with breast, ovarian, and cervical cancers. It may assist clinicians in formulating an effective treatment plan. Notably, false-positive lymph nodes were identified on ^18^F-FDG PET/CT in all three cancer types, whereas none were detected by ^68^Ga-FAPI PET/CT. These findings suggest that ^68^Ga-FAPI PET/CT can help identify false-positive lymph nodes previously detected by ^18^F-FDG PET/CT, thereby reducing unnecessary biopsies and complications. Nonetheless, ^68^Ga-FAPI PET/CT represents a promising imaging modality, particularly when ^18^F-FDG PET/CT is of limited utility.

This study has several limitations. First, the sample size was relatively small (*n* = 35) and heterogeneous, consisting of patients with breast, ovarian, and cervical cancers. Second, the representation of various malignancies was imbalanced, necessitating prospective trials with larger patient populations to further evaluate the diagnostic efficacy of this approach. Third, FAPI uptake may be influenced by hormonal factors in normal hormone-responsive organs or by the menstrual cycle.

## Conclusion

In conclusion, our results indicate that ^68^Ga-FAPI PET/CT demonstrates higher tracer uptake and is partially superior to ^18^F-FDG PET/CT for detecting primary and metastatic lesions in patients with common gynecological malignancies. Additionally, it yielded favorable results in the initial assessment, detection of recurrences, and differentiation of false-positive lymph nodes. ^68^Ga-FAPI PET/CT emerges as a highly promising tracer and may serve as a valuable supplement to ^18^F-FDG PET/CT. However, larger prospective studies are needed to confirm this.

## Data Availability

The datasets used and/or analysed during the current study are available from the corresponding author on reasonable request.

## Abbreviations

^18^F-FDG: Fluorine-18-fluorodeoxyglucose
^68^Ga-FAPI: Gallium-68-fibroblast activation protein inhibitor
PET: Positron emission tomography
CT: Computed tomography
SUV_max_: Maximum standardized uptake values
